# Risk factors for esophageal fistula in thoracic esophageal squamous cell carcinoma invading adjacent organs treated with definitive chemoradiotherapy: a monocentric case-control study

**DOI:** 10.1186/s12885-018-4486-3

**Published:** 2018-05-18

**Authors:** Takeshi Kawakami, Takahiro Tsushima, Katsuhiro Omae, Hirofumi Ogawa, Hiromichi Shirasu, Yosuke Kito, Yukio Yoshida, Satoshi Hamauchi, Akiko Todaka, Nozomu Machida, Tomoya Yokota, Kentaro Yamazaki, Akira Fukutomi, Yusuke Onozawa, Hirofumi Yasui

**Affiliations:** 1 Division of Gastrointestinal Oncology, Shizuoka Cancer Center, Shizuoka, Japan; 2 Clinical Research Promotion Unit, Shizuoka Cancer Center, Shizuoka, Japan; 3 Division of Radiation Oncology, Shizuoka Cancer Center, Shizuoka, Japan; 4 Division of Clinical Oncology, Shizuoka Cancer Center, Shizuoka, Japan; 5 Division of Gastrointestinal Oncology, Shizuoka Cancer Center, 1007 Shimonagakubo, Nagizumi-cho, Sunto-gun, Shizuoka, 411-0934 Japan

**Keywords:** Esophageal fistula, Esophageal squamous cell carcinoma, Chemoradiotherapy, Risk factor

## Abstract

**Background:**

Standard treatment for unresectable esophageal squamous cell carcinoma (ESCC) without distant metastasis is definitive chemoradiotherapy (dCRT), in which the incidence of esophageal fistula (EF) is reported to be 10–12%. An ad hoc analysis of JCOG0303, a phase II/III trial of dCRT for patients with unresectable ESCC (including non-T4b), suggested that esophageal stenosis is a risk factor for EF. However, risk factors for EF in patients limited to T4b ESCC treated with dCRT have yet to be clarified. The aim of this study was to investigate risk factors for EF in T4b thoracic ESCC treated with dCRT.

**Methods:**

We retrospectively analyzed the data of consecutive T4b thoracic ESCC patients who were treated with dCRT (cisplatin and fluorouracil) at Shizuoka Cancer Center between April 2004 and September 2015.

**Results:**

Excluding 8 patients with esophageal fistula clearly attributable to other iatrogenic interventions, the data of 116 patients who met the inclusion criteria were analyzed. Esophageal fistula was observed in 28 patients (24%). Although the fistula was closed in 5 patients, overall survival was significantly shorter in patients who experienced esophageal fistula (8.0 vs. 26.8 months; *p* < 0.0001). Among four potential variables extracted in univariate analysis, namely, total circumferential lesion, elevated CRP level, elevated white blood cell count, and anemia, the first two were revealed as risk factors for esophageal fistula in multivariate analysis.

**Conclusions:**

This study demonstrated that total circumferential lesion and CRP ≥1.00 mg/dL are risk factors for esophageal fistula in T4b thoracic ESCC treated with dCRT.

**Trial registration:**

This study was retrospectively registered.

**Electronic supplementary material:**

The online version of this article (10.1186/s12885-018-4486-3) contains supplementary material, which is available to authorized users.

## Background

Standard treatment for unresectable esophageal squamous cell carcinoma (ESCC) without distant metastasis is definitive chemoradiotherapy (dCRT) with 5-fluorouracil plus cisplatin, using a total irradiation dose of 50.4 or 60 Gy [1–6]. While dCRT improves prognosis [5], it is sometimes associated with the life-threatening adverse event of esophageal fistula. The incidence of esophageal fistula was reported to be 10–29% in patients receiving dCRT for ESCC [7, 8] and the prognosis of patients harboring esophageal fistula, especially arterio-esophageal fistula, is poor [9]. ESCC invading adjacent organs is known to be associated with a high incidence of esophageal fistula [10]; however, the risk factors for this have remained unclear. Recently, an ad hoc analysis of JCOG0303, which compared the efficacy of low-dose and standard-dose chemoradiotherapy for unresectable ESCC invading adjacent organs and/or distant metastasis, suggested that esophageal stenosis is a risk factor of esophageal fistula [7]. However, this study had some weaknesses, such as regarding patient selection and the definition of the risk factors. For example, patients whose primary tumor had not invaded adjacent organs were also included. However, given the low incidence of esophageal fistula formation upon treatment with dCRT for non-organ-invading tumors, this condition is not clinically relevant. This group also did not specifically define esophageal stenosis. Instead, they judged esophageal stenosis subjectively from clinical symptoms and/or patient reports. Therefore, we conducted this retrospective study to perform more precise investigation of risk factors for esophageal fistula in ESCC invading adjacent organs treated with dCRT.

## Methods

### Patients

We retrospectively collected the data of consecutive ESCC patients who were treated with dCRT with cisplatin and fluorouracil at Shizuoka Cancer Center between April 2004 and September 2015. All data were collected from electronic medical records. All procedures were in accordance with institutional and national standards on human experimentation, as confirmed by the ethics committee of Shizuoka Cancer Center, and also with the Declaration of Helsinki of 1964 and later versions. Inclusion criteria were as follows: (1) age over 20 years; (2) Eastern Cooperative Oncology Group (ECOG) performance status (PS) 0–1; (3) depth T4b in accordance with the UICC-TNM classification 7th edition; (4) primary lesion located in the thoracic esophagus; (5) no distant organ metastasis other than supraclavicular lymph node metastasis; (6) no esophageal fistula before dCRT; (7) creatinine clearance ≥50 mL/min; and (8) other organ functions preserved.

All blood data used in the analysis were taken within 14 days before the initiation of dCRT. T4b was diagnosed by radiologists, oncologists, and surgeons based on findings of enhanced CT. Aortic invasion was defined as a contact angle of esophageal cancer to the aorta of greater than 90°, and tracheal or bronchial invasion was defined as deformities of the trachea or bronchi due to contiguous cancer. Esophageal fistula was defined as a connection between the esophagus and adjacent organs detected by CT, endoscopy, or X-ray with diatrizoate meglumine and diatrizoate sodium solution. We defined the characteristic clinical manifestations of esophageal fistula, such as a dramatic increase of sputum or massive hematemesis, as esophageal fistula if it was impossible to evaluate esophageal fistula by imaging studies.

An Olympus H260® endoscope is routinely used for endoscopic examination of the upper gastrointestinal tract at our institution. Here, we defined esophageal stenosis as cases in which it was impossible to pass an endoscope through the lesion. The diagnosis of total circumferential lesions was made only after confirmation of the total circumferential connection of lesions. Endoscopists used an ultrathin endoscope (e.g., Olympus XP260®) if an Olympus H260® scope could not pass through the lesion.

All patients provided written informed consent before the start of treatment.

### Treatment

The dCRT regimen followed the standard-dose cisplatin plus fluorouracil regimen of JCOG0303 [4]. Chemotherapy consisted of intravenous infusion of 70 mg/m^2^ cisplatin with adequate hydration on days 1 and 29, and continuous infusion of 700 mg/m^2^ fluorouracil on days 1–4 and 29–32. For an antiemetic, the infusion of dexamethasone and palonosetron hydrochloride along with oral or infused aprepitant was used, after they had been approved for antiemesis. Additional cisplatin plus fluorouracil doublet therapy was continued as necessary after dCRT. RT was planned to deliver a total of 60 Gy/30 Fr using a linear accelerator with a 6-, 10-, or 18-MV photon beam. Three-dimensional dose calculations were performed using Pinnacle3 software (ADAC, Milpitas, CA) with correction for tissue-density inhomogeneity. The treatment planning was based on 3.8- to 5-mm-thick CT scans obtained in the treatment position. The gross tumor volume was based on clinical examinations including CT scan and endoscopy. The clinical target volume (CTV) for the primary tumor was created to add a 2-cm margin craniocaudally to account for subclinical tumor extension. A CTV margin for metastatic lymph nodes was not added. Elective nodal irradiation was not performed. The planning target volume (PTV) was created to add 0.5–1 cm for lateral margins and 1–1.5 cm for craniocaudal margins. The radiation fields were designed to cover the PTV with an adequate margin. A dose of 60 Gy to the center of the PTV was prescribed. The dose to the spinal cord was kept at ≤45 Gy. The mean doses of the heart and the volume of the lung receiving 20 Gy (V20) were kept at ≤40 Gy and ≤ 35%, respectively.

### Statistics

Fisher’s exact test and the Mann–Whitney U test were used for comparison between patients who experienced esophageal fistula (EF+) and those who did not (EF−). Univariate and multivariate analyses were carried out using logistic regression to estimate the odds ratio (OR). In the multivariate analysis, a standard stepwise method was adopted for the selection of informative risk factors. Overall survival (OS) was calculated from the date of initiation of dCRT until death from any cause. Patients who were alive or whose data were missing at the data cut-off were censored. Time to esophageal fistula was calculated from the date of initiation of dCRT until esophageal fistula. Patients who died before fistula were censored. Survival time was calculated by the Kaplan–Meier method. A *p*-value of < 0.05 was considered statistically significant. All analyses were conducted using R statistical software version 3.3.2.

## Results

### Patient characteristics

After excluding 8 patients with EF clearly attributable to medical intervention, such as endoscopic dilatation, the data of 116 patients who met the inclusion criteria were analyzed. Esophageal fistula was observed in 28 patients (24%). Median age (range) was 65 (41–80) years, median tumor size (range) was 70 (12–200) mm, total circumferential lesion was present in 60 patients, and esophageal stenosis was present in 74 patients. Patients’ characteristics in the EF+ group vs. those in the EF− group were as follows: median age (range), 62 (41–74) vs. 65 (47–80) years; ECOG PS 0/1, 19/9 vs. 58/30 patients; median tumor size (range), 80 (12–110) vs. 70 (20–200) mm; total circumferential lesion, 20 vs. 40 patients; esophageal stenosis, 18 vs. 56 patients; aortic invasion, 16 vs. 38 patients; tracheal or bronchial invasion, 24 vs. 68 patients; and CRP ≥1.00 mg/dL, 19 vs. 29 patients, respectively (Table 1).

**Table 1:** Patients’ characteristics

Characteristics	Number of patients (*N* = 116)
Median age (range), years	65 (41–80)
Sex, male	102
ECOG PS 0/1	77/39
Location of primary lesion Ut/Mt./Lt	46/66/4
Median tumor size (range)^a^, mm	70 (12–200)
Total circumferential lesion	60
Esophageal stenosis	74
Invading adjacent organs	
Aorta	54
Trachea or bronchus	92
Macroscopic type	
Ulcerative type	113
WBC ≥ 10,000/μL	15
Hb < 12 g/dL	19
Alb < 3.5 g/dL	7
CRP ≥ 1.00 mg/dL^b^	48

Abbreviations: *ECOG* eastern cooperative oncology group, *PS* performance status, *Ut* upper thoracic esophagus, *Mt.* middle thoracic esophagus, *Lt* lower thoracic esophagus, *WBC* white blood cell, *Hb* hemoglobin, *Alb* albumin, *CRP* C reactive protein

^a^Data missing for one patient

^b^Data missing for two patients

### Risk factors for esophageal fistula

In univariate analysis, total circumferential lesion [OR 3.00; 95% confidence interval (CI) 1.19–7.54; *p* = 0.019], WBC ≥10,000/μL (OR 3.33; 95% CI 1.08–10.2; *p* = 0.036), Hb < 12.0 g/dL (OR 3.69; 95% CI 1.32–10.4; *p* = 0.013), and CRP ≥1.00 mg/dL (OR 4.15; 95% CI 1.67–10.3; *p* = 0.002) were significantly different between the EF+ group and the EF− group (Table 2). Total circumferential lesion (OR 3.09; 95% CI 1.14–8.39; *p* = 0.027) and CRP (OR 5.19; 95% CI 1.93–13.9; *p* = 0.001) were confirmed as independent risk factors for esophageal fistula in multivariate analysis (Table 3).

**Table 2:** Univariate analysis for the incidence of esophageal fistula

Characteristics	EF+ (*N* = 28)	EF− (*N* = 88)	OR	95% CI	*P*-value
Age ≥ 65 years	13	47	0.76	0.32–1.77	0.520
Sex, male	23	79	0.52	0.16–1.72	0.286
ECOG PS 0/1	19/9	58/30	0.92	0.37–2.27	0.849
Tumor size ≥70 mm^a^	19	47	2.07	0.82–5.23	0.123
Total circumferential lesion	20	40	3.00	1.19–7.54	0.019
Esophageal stenosis	18	56	1.03	0.42–2.50	0.950
Aorta invasion	16	38	1.75	0.74–4.14	0.200
Tracheal or bronchial invasion	24	68	1.76	0.55–5.69	0.341
WBC ≥ 10,000/μL	7	8	3.33	1.08–10.2	0.036
Hb < 12 g/dL	9	10	3.69	1.32–10.4	0.013
Alb < 3.5 g/dL	4	3	4.72	0.99–22.6	0.052
CRP ≥ 1.00 mg/dL^b^	19	29	4.15	1.67–10.3	0.002

Abbreviations: *EF* esophageal fistula, *OR* odds ratio, *CI* confidence interval, *WBC* white blood cell, *Hb* hemoglobin, *Alb* albumin, *CRP* C reactive protein

^a^Data missing for one patient

^b^Data missing for two patients

**Table 3:** Multivariate analysis for the incidence of esophageal fistula

Characteristics	EF+ (*N* = 28)	EF− (*N* = 88)	OR	95% CI	*P*-value
Total circumferential lesion	20	40	3.09	1.14–8.39	0.027
Aorta invasion	16	38	2.56	0.97–6.75	0.058
CRP ≥ 1.00 mg/dL^a^	19	29	5.19	1.93–13.9	0.001

Abbreviations: *EF* esophageal fistula, *OR* odds ratio, *CI* confidence interval, *CRP* C reactive protein

^a^Data missing for two patients

### Overall survival and event-free survival

**Fig. 1:**
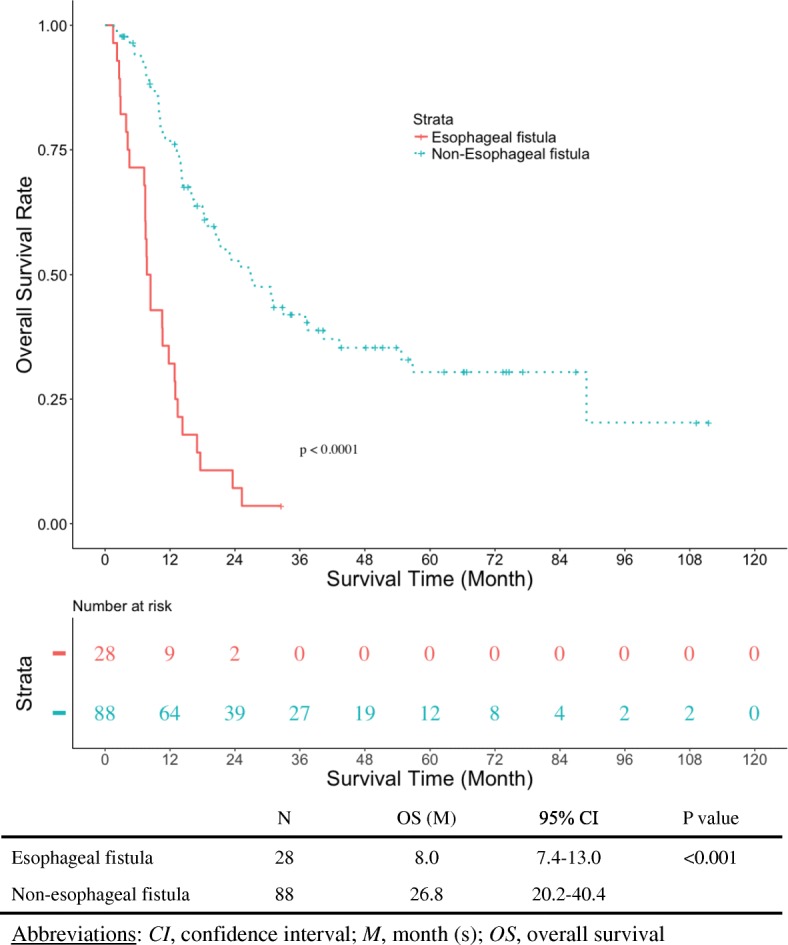
Kaplan-Meier curve for overall survival (OS). OS in the esophageal fistula group was significantly shorter than that in the non-esophageal fistula group (8.0 vs. 26.8 months; *p* < 0.001)

**Fig. 2:**
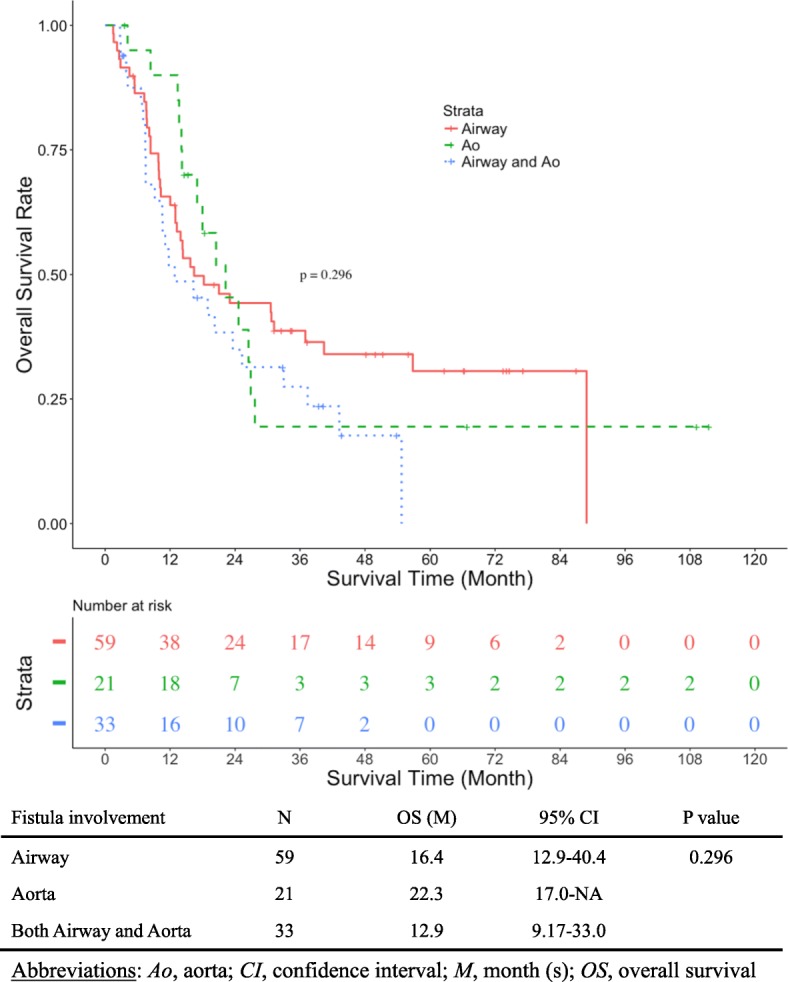
Kaplan-Meier curve for overall survival (OS) according to fistula involvement. There was no significant difference in OS according to fistula involvement (Airway vs. Aorta vs. Both Airway, 16.4 vs. 22.3 vs. 12.9 months; *p* = 0.296)

With a median follow-up time of 40 months in censored cases, OS in the EF+ group was significantly shorter than in the EF− group (8.0 vs. 26.8 months; *p* < 0.0001) (Fig. 1). Median time to esophageal fistula in the EF+ group was 2.5 months (95% CI 2.0–4.0 months). Only one patient survived and received subsequent chemotherapy at the data cut-off point. There was no difference in overall survival among EF+ patients whose fistula involvement was airway, aorta, or both (16.4 vs. 22.3 vs. 12.9 months; *p* = 0.296) (Fig. 2).

### Clinical course of 28 patients with esophageal fistula

Esophageal fistula occurred in 5 patients during dCRT and in 23 patients after it. Five patients experienced closure of the esophageal fistula during dCRT or subsequent chemotherapy. Among these 28 patients, 8 died because of massive hemorrhage and 8 because of infection related to the esophageal fistula. Seven patients died due to progression of ESCC and four patients due to other factors. Eight patients died within 30 days after esophageal fistula. Only one patient was alive at the data cut-off point.

### Post-dCRT treatment in 28 patients with esophageal fistula

Seventeen patients who experienced esophageal fistula received best supportive care without chemotherapy/radiotherapy after the formation of the fistula. Ten patients received the following chemotherapy: cisplatin plus fluorouracil in 7 patients, nedaplatin plus vindesine in 1 patient, docetaxel in 1 patient, and paclitaxel in 1 patient. One patient underwent salvage surgery without subsequent chemotherapy.

## Discussion

In the current study, the risk factors of esophageal fistula for T4b ESCC were total circumferential lesion and CRP ≥1.00 mg/dL. To the best of our knowledge, no definitive risk factors for esophageal fistula formation have been identified in esophageal cancer patients receiving dCRT, despite esophageal fistula being fatal, as previously reported [9, 10].

A supplemental analysis of JCOG0303 identified esophageal stenosis as a risk factor for esophageal fistula [7], although this was not found to be a risk factor under the definition used in the current study. However, there were some differences between the previous study and the present study. First, regarding the backgrounds of the subjects, better selection was performed in this study than in JCOG0303: JCOG0303 included patients with non-T4b ESCC, while those included in this study were limited to T4b ESCC. Therefore, we specifically investigated risk factors of esophageal fistula in T4b ESCC in this study, which is clinically relevant. A second difference between the studies was that, in JCOG0303, the definition of esophageal stenosis was not described, so it might have differed depending on the clinical investigator. In contrast, we defined esophageal stenosis as cases in which the endoscope could not pass through the lesion. This enabled us to perform a more precise evaluation of risk factors of esophageal fistula only in T4b ESCC patients.

CRP ≥1.00 mg/dL was also a risk factor for esophageal fistula in this study. This may reflect tissue damage induced by an elevated level of IL-6, which causes tissue inflammation [11]. Albumin concentration is usually decreased when IL-6 is overproduced; however, there were only a few patients with a low albumin concentration among the study population. A possible explanation for this is that T4b ESCC patients generally do not eat well and are often dehydrated. In this study, we did not measure the IL-6 level, so we could not verify this hypothesis.

Even patients with such risk factors are not a contraindication for dCRT because they have a chance of achieving a complete response, which could prolong the survival time [4]. In fact, five patients with esophageal fistula experienced fistula closure by continuing the treatment and had survived for more than 1 year at the data cut-off. However, overall survival of the EF+ group was significantly shorter than that of the EF− group in the current study. Therefore, the development of a treatment strategy for ESCC patients with a risk of fistula formation is warranted.

Recently, some reports have suggested that induction chemotherapy followed by dCRT reduces the incidence of esophageal fistula [12, 13]. The reported esophageal fistula rate was in the range of 3–6%, and one of these studies showed that this rate in the group with induction chemotherapy followed by CRT was significantly lower than in those with CRT alone (6% vs. 17%, *p* = 0.0379) [12]. Therefore, induction chemotherapy followed by CRT may be a feasible treatment strategy for T4b thoracic ESCC with such risk factors.

The present study has several limitations. First, this is a retrospective study with a small sample size from a single institution. Second, it was difficult to distinguish treatment-related esophageal fistula and disease progression precisely. Third, bronchoscopy was not performed routinely to diagnose esophageal fistula before dCRT. Finally, we selected all fistula cases in the study population regardless of the duration from the commencement of dCRT; therefore, some cases with esophageal fistula might have been due to progressive disease. Considering these limitations, we particularly focused on T4b disease and used a precise definition of esophageal stenosis, so that we could reveal reliable risk factors for esophageal fistula formation in T4b ESCC in the current study.

## Conclusions

This study suggested that total circumferential lesion and elevated CRP level are risk factors for esophageal fistula in T4b thoracic ESCC treated with dCRT. We believe that these results warrant validation in a future prospective investigation.

## Additional file

Additional file 1: dataset. This dataset was used for analysis of patients’ characteristics, calculating survival time by Kaplan-Meier methods, and estimating the odds ratio for the incidence of esophageal fistula in multivariate analysis. (XLSX 23 kb)

## Abbreviations

Alb: Albumin
CI: Confidence interval
CRP: C-reactive protein
CTV: Clinical target volume
dCRT: definitive chemoradiotherapy
ECOG: Eastern Cooperative Oncology Group
ESCC: Esophageal squamous cell carcinoma
Hb: Hemoglobin
OR: Odds ratio
PS: Performance status
PTV: Planning target volume
WBC: White blood cell
